# Enhanced convolutional neural network accelerators with memory optimization for routing applications

**DOI:** 10.1371/journal.pone.0320813

**Published:** 2025-04-21

**Authors:** Srikanth Prasad Nallabelli, Sundar Sampath

**Affiliations:** School of Electronics Engineering, Vellore Institute of Technology, Vellore, Tamil Nadu, India; New York University Abu Dhabi, UNITED ARABEMIRATES

## Abstract

Currently, Convolutional Neural Networks (CNN) accelerators find application in various digital domains, each highlighting memory utilization as a significant concern leading to system degradation. In response, our present work focuses on optimizing the memory usage of CNN through a strategic approach. The resulting system is coined as the Memory Optimized Zebra CNN (MOZC). In the initial stage, the CNN accelerator is constructed with optimized features, specifically addressing the network routing function. In this context, our approach draws inspiration from zebras, aiming to identify the shortest path between network nodes. The Field-Programmable-Gate-Arrays (FPGA) are employed for evaluating MOZC performance, considering parameters such as lookup table (LUT), Flip-Flop (FF), memory utilization, power consumption, Digital-Signal Processing (DSP), and Giga-Operations-Per-Second per watt (GOPS/W). Additionally, key parameters like data delivery and Throughput assess routing and data transmission robustness. Video data is utilized to determine routing efficiency, and the achieved highest GOPS/W is recorded as 30.43, marking a substantial improvement over conventional CNN accelerators.

## 1 Introduction

CNNs are widely used in image and prediction systems because they can process vast amounts of data through multiple layers [1]. However, the substantial computational demands of CNNs present significant challenges in managing and storing data within the limited on-chip memory available on embedded systems [2]. Memory computing architectures can optimize power efficiency at CNN applications, but they may have computation and data handling mismatches. In large-scale CNN, data often needs to be fetched from off-chip memory due to the insufficient capacity of on-chip memory to store all required parameters and intermediate results structure from the off-chip memory location [3]. The collected data is stored in the embedded memory system [4]. To execute many operations in parallel, the CNN allows efficient parallelism [5]. The structure of CNNs will enable them to have efficient parallel modelling where convolutional layers can independently handle multiple regions of input data. This allows various operations, such as convolutions, activations, and pooling, to be executed parallelly. This inherent parallelism aligns well with hardware acceleration, making CNN well-suited for high-performance applications despite the substantial computational demands. While executing a CNN on 720p video includes large amounts of convolutions. 3.4 billion memory access is required for a single layer [6].The number of external accesses can be decreased by data reuse on-chip memory. For implementing CNN with sufficient bandwidth, high-speed floating point, and high parallelization, the graphics processing unit (GPU) is used [7].

CNN computations are typically very computationally expensive; GPUs and FPGAs are often employed to accelerate the algorithms. GPUs utilize many cores to process multiple data points in parallel, enabling efficient CNN computation. This parallelization occurs independently of batch size, as the GPU cores handle numerous operations simultaneously. The cost of CNN is high because of its computational complexity [8]. FPGAs provide customized hardware to optimize memory access patterns. In recent years, CNN accelerators have been used for high-performance digital systems and low-power FPGA [9]. FPGA cannot fully store a huge-scale CNN model in the on-chip memory resources. Hence, FPGA implementation on CNN off-chip memory is used to overcome this problem but does not provide application flexibility [10]. The most useful CNN models for visual representation are imposed on evaluation without off-chip memory. The proposed CNN accelerators perform well since they are employed in an FPGA-only on-chip memory [11].

A reconfigurable optimized memory computing or normalized memory computing accelerator supporting high resource analysis with different kernel sizes [12]. It reduces off-chip memory interactions, decreasing bandwidth constraints and energy usage. They can meet the varying needs of various neural network models while maintaining high Throughput by using on-chip memory for data storage and processing.[13]. This method dramatically improves the performance of visual categorization tasks by lowering latency and energy overhead, providing a more scalable and resource-efficient alternative to typical central processing units (CPU) based designs [14]. The main contribution of on-chip memory storage lies in its utilization across several neural network models. Off-chip bandwidth remains a critical bottleneck for visual classification tasks [15]. In hardware architecture, CPUs face various challenges, such as limited memory bandwidth, high memory access latency, and significant energy consumption due to large-scale data communication [16]. The GPU has to be applied widely to the consequence and deployment of CNN [17]. The succeeding layer of architecture appraises the historical accomplishment of CNN.

As CNN develops more complex, the time required to compute CNN models restricts its use. Convolutional computation has been discovered to be the most time-consuming part of CNN [18]. As a result, numerous neural network accelerators, such as GPU, FPGA, and application-specific integrated circuits (ASICs), have been developed to speed up the process [19]. FPGAs have emerged as a favourable solution for implementing CNN accelerators due to their flexibility and ability to customize memory access patterns, significantly enhancing performance while reducing power consumption [20]. These advancements have led to implementation of the CNN accelerator, which can be easily modified to accommodate varying configurations, such as the number of layers and neurons [21].The design and benchmarking of CNN rely on progress in both hardware and software domains. Typically, the network layers are evaluated one by one, i.e. each layer is analyzed and trained separately in a sequence over time [22]. This step-by-step approach assists in obtaining slightly better performance and optimizing incrementally [23]. Hence, the strategy aims to solve problems of deploying CNN on embedded devices, like the small available amount of local memory, the requirement for efficient access patterns in memory, and high computational complexity.

FPGA-based implementations are particularly appealing for resource-constrained applications, enabling high-performance digital systems that can adapt to varying configurations. It employs an innovative memory-optimized zebra CNN (MOZC) accelerator to optimize memory. It utilises dynamic source routing (DSR) for data broadcast and CPU performance optimization for the best use of on-chip resources, improving performance. ZO is an efficient algorithm that solves complex multi-objective optimization problems, such as memory usage and routing efficiency. When combined with CNN in the MOZC framework, ZO enhances CNN’s ability to fine-tune architecture for optimal performance. The ZO algorithm discovers the best combination of CNN by adjusting memory consumption and computational effectiveness. It iteratively tunes the parameters of the CNN, such as layer depth, filter sizes, and pooling strategies, to find an optimal trade-off. In this process, the CNN can adaptively allocate resources and reduce memory usage without compromising routing efficiency. By making use of the dynamic and adaptive characteristics of ZO, MOZC ensures that the CNN MOZC ensures that CNN operates efficiently in memory, maintaining its accuracy and speed in routing decisions, making it a suitable approach.

The key contribution of this study is described as follows

- Initially, a novel MOZC accelerator has been designed with the needed functional parameters.
- Consequently, the DSR protocol was implemented for the robustness analysis, and the shortest routing was established using the fitness function in the MOZC strategy.
- Moreover, the data broadcasting process was initiated to validate the stability performance of the designed framework.
- Henceforth, the CPU performance was analyzed and validated in two phases, before and after applying the optimization function.
- At last, the performance parameters were calculated and compared with other traditional models.

The presented research study has been arranged in second section, which deals with traditional approaches in the network accelerator with merits and demerits; the Third section deals with the traditional CNN accelerator system with problems. Moreover, the fourth section describes the newly introduced model for the CNN accelerator performance improvement. The fifth section justifies the implemented model with results and comparisons. Finally, the research discussion concludes in the sixth section.

## 2 Literature review

Yuan, Tian, et al. [24] developed a hardware-oriented constrict strategy for CNN. In this method, the deep nets CNN is classified into different neurons, such as filtering, dense, and Output. The hardware accelerator design can be tested for image detection applications and several real-time resources. This approach cannot be used when many operations are merged in parallel decoding.

Juracy et al. introduced an accurate, quick, and extensive power performance parameter appraisal of the CNN hardware accelerators [25].In this method, the CNN is integrated with the TensorFlow framework. The analytic approach reduces errors in calculation. Area, power, energy, and memory accesses are estimated using the analytical model. It faces challenges in complexity due to the integration of these approaches.

The metaheuristics methodology was carried out by Sait, Sadiq M., et al. [26]. This implementation utilizes the optimal infrastructure floor plan to build a robust multimodel deep network accelerator. At the same time, the multi-parameter deep network accelerator has been implemented with the CNN principles. Due to the default parameter change, the built Accelerator met the complexity in processing the input features for a suitable outcome. The recorded complexity is in training and weight updating of the input data.

Rios et al. [27] have built bit-wise operations with the parallel infrastructure to regulate the memory processing function. Hence, this bit-wise operation is supported by the hardware component logic units. It gives the proper validation outcome in processing the mathematical hardware models through dimensionality reduction and weight updating. However, the bit-wise processing approach is unsuitable for the extensive data importing process and applications.

In this paper, Kim et al. [8] Kim was presented an expensive CNN as the actual Traffic Sign detection component. Further, this predictive system contains the optimized constraints for hardware and software-level programs. These hardware accelerators improve computation performance and framework for quantization, full integer-based inference, and floating-point training. The BN fusion method is used to reduce the computational overhead.

Conficconi et al. [28] have developed an open-source hardware-software framework to create a flexible architecture for performing the computation of similarity metrics, which is the most computationally demanding aspect of registration methodologies. The developed architecture’s design options are investigated and validated on many FPGAs using various combinations of the design parameters. Though this framework yields encouraging results in speedup and power efficiency, it does not address memory optimization challenges in broader compute-intensive tasks.

Wu et al. [29] have developed a novel compressive depth reconstruction design strategy for the least absolute shrinkage and selection operator (LASSO) problem, which employs a compact ADMM solver and adjusts accuracy scaling in an iterative optimisation process. The design focuses heavily on hardware resources and power savings but does not extensively explore the impact on overall computation.

AZZAZ et al. [30] have described implementing morphological image techniques such as dilation, erosion, and linear filtering. This is executed on a Zedboard FPGA platform with a Zynq device linked to a PC via an Ethernet connection. Experiments show that the suggested strategy is feasible and can be expanded for further use. Still, the limitation is that it may not fully leverage the potential performance optimizations available in low-level hardware design, leading to suboptimal speed or resource usage.

Andrey Kvochko et al. [31] Off-chip storage is frequently considered significant enough to store all of the network’s objects, inputs, outputs, and eventual results that might arise while the model is executed. Given that most edge devices, like smartphones, have memory use caps, this assumption might not apply to them. Thus, a revolutionary method of reducing the amount of off-chip memory used by a neural network utilizes a tile-aware allocator to reuse memory used by portions of a tensor. In contrast, the tensor as a whole expires. It outlines the requirements for this kind of off-chip memory allocation strategy. It presents the findings, demonstrating that it can reduce maximum off-chip memory utilization by up to 33% in typical network designs.

The non-volatile memory-based CNN accelerator was studied by Lixia Han et al. [32]. Here, a mapper is created to optimize the weight transformation, pipeline, division, and allocation of CNN simulations to memory computing chips. Moreover, precision and performance evaluators are constructed in tandem to figure out overheads related to energy, delay, precision, and area while considering design constraints at various levels. The algorithmic adaptor retrains CNN parameters using energy-aware and nonideal-aware instructions. It results in improved hardware accuracy within a limited energy restriction. As a result, the CNN prediction rate increased, but the memory was not optimized.

Yen-Wen chen et al. [33] have implemented a dynamic hybrid accelerator based on the CNN principles. Here, mixed precision is introduced to analyze and regulate the noisy features in the input data. The key objective of this RAM-based Accelerator is to optimize the time needed to predict other application constraints. Finally, the computation period of the memory accelerator was reduced. However, the accelerator is not fully optimized and requires further enhancements for better performance.

Yu-Pei Liang et al. [34] have described that CNN reduces computing time and energy consumption through its organizational structure. CNN’s actual time capabilities are demonstrated by its complete calculation being finished in 330,000 phases with only 29k artificial neural network variables. The suggested architecture significantly reduces the amount of cycles required for hardware computations. Additionally, this work adds Gaussian white noise to the vibrating signal collection so that noise ratio evaluation can be performed. A noisy learning set is appended to the primary information for developing neural networks to increase precision. In conclusion, at a clock speed of 100 MHz, the post-layout modelling of the suggested design enables real-time defect diagnosis with a resolution of 95.31% and an overall power utilization of 65.608 mW.

Although the literature gives essential insight into different designs of CNN accelerators, the proposed MOZC has features that make it unique from existing ones. MOZC does not optimize the CPU hardware infrastructure or off-chip memory system, but it focuses on memory optimization for CNN accelerator by leveraging Zebra optimization. This accommodates memory constraints and improves the efficiency of the data traffic routing mechanism in the network. This means the CNN accelerator remains robust and uses less memory than previous methods.

## 3 System design with problem

Memory optimization is critical for enhancing digital systems’ performance, especially during resource-intensive processes. It improves system resource management by reducing delay and ensuring smooth operation during intensive processing. Considering this need, this study focuses on memory optimization with a routing application used to assess the robustness of the implemented CNN accelerator.

**Fig 1:**
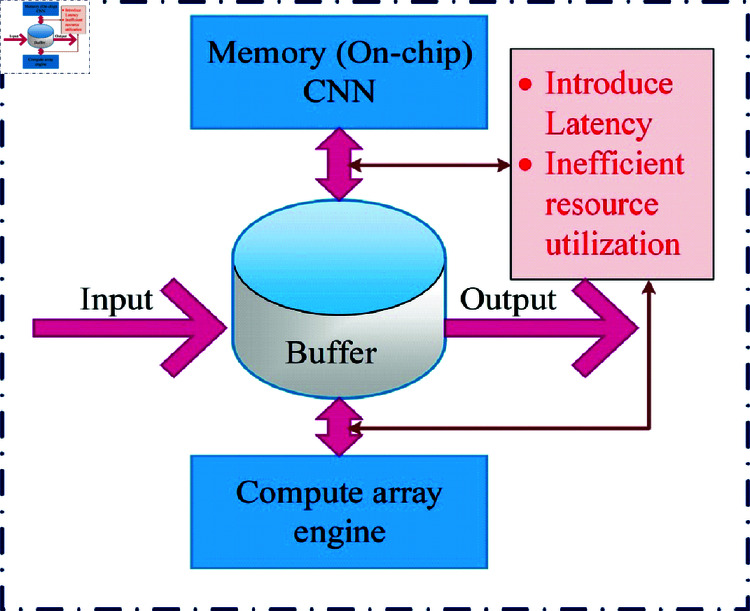
Drawbacks of CNN accelerator.

Data movement between the memory and compute engine lowers performance due to Latency. The compute array engine might not be efficient for certain CNN operations, causing a waste of resources and high power consumption. Usually, the CNN architecture needs more time and resources to run the system due to several features and limited running capacity. It might slow down the connected application and maximize the system requirement. Its demerits are defined in Fig 1. Considering this; the present article intends to develop an optimized CNN to reduce CPU memory usage and enrich the routing performance.

## 4 Proposed methodology

A novel memory-optimized zebra CNN (MOZC) accelerator was introduced in this proposed article to enhance the network routing mechanism. Here, the chief intent of this study is to optimize CPU memory usage by improving the CNN accelerator. Hence, zebra optimization [35] is obtained to tune the accelerator performance. Thus, the optimized CNN accelerator is named as MOZC. In addition, the network routing mechanism was adopted to check the working robustness of the designed CNN accelerator, and the data-sharing process was executed.

**Fig 2:**
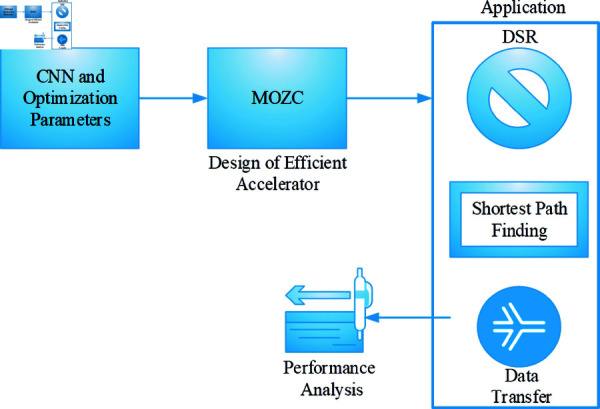
Proposed architecture.

DSR protocols [36] are a type of routing protocol. It enables nodes to select the shortest path for their data packets by evaluating prevailing network situations. The main module in DSR is source routing, which means the path from a packet is discovered by its origin node and placed in the header of this package. It enables effective routing in unpredictable environments where network topology changes often. It is implemented to perform the network routing. In the routing mechanism, MOZC is utilized to find the shortest path. Finally, the parameters were calculated and compared with other models. The proposed architecture is elaborated in Fig 2.

The proposed MOZC approach improves the efficiency of CNN accelerators, especially with CPU memories. It accomplishes this by blending in the zebra optimization approach. It optimizes accelerator performance by adjusting key parameters such as weight updates and operational steps under different CNN layers. In particular, the zebra optimization algorithm is used to learn the relative connections between each hidden layer, subsequently increasing memory management and computational capabilities. Furthermore, a network routing mechanism has been integrated to guarantee stable data flow and prove the effectiveness of an accelerator. Using these approaches, MOZC significantly reduces memory usage in CNN without compromising speed and accuracy.

### 4.1 Process of proposed MOZC methodology

The MOZC combines a hardware accelerator and a Zebra optimization-based tuning method that enhances CNN accelerator performance. The zebra optimization method adjusts the CNN weights and operation parameters for memory-efficient processing. The prime process of any neural system is the data initialization or the training function, defined in Eq 1. According to that, the data initialization of the novel MOZC was activated in the initial phase. Where *X* determines the input data and the Importing process are exposed *I*(*X*). Moreover, the *n* number of input samples is described as 1,2,3,...,*n*.


$$\begin{array}{ccc} I(X)=T\{X[1,2,3,\ldots ,n]\} & & \end{array}$$ (1)

The data importing function is modelled, and the operational steps have been designed with the associated layer count of the CNN. Here, *T* denotes the training variable, *X* denotes the feature set of an individual input sample. It represents the input data process through layer processing and dimensionality reduction as part of training. Moreover, the appropriate weights of the trained input are substituted in the MOZC hidden layer. Henceforth, the data is prepared for further functional processing, and dimensionality reduction and duplicate elimination are performed in the pooling layer by analyzing the weight updates. Hence, Eq 2 performs the weight update from the zebra solution. .


$$\begin{array}{ccc} W=\text{crossover}(W_{i}^{a},W_{i}^{c}) & & \end{array}$$ (2)

**Fig 3:**
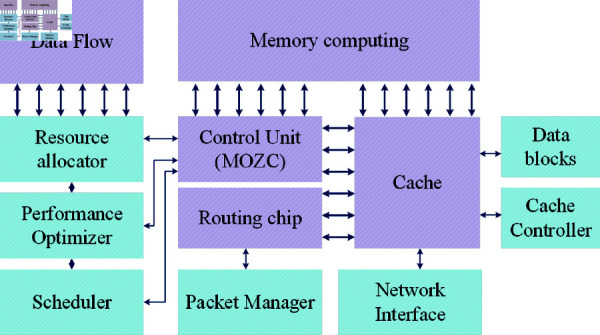
Architecture of MOZC.

Here, $W_{i}^{a}$ determines the connection and position of primary nodes and $W_{i}^{c}$ defines the connection and position of the following nodes from the primary location. The weight update mechanism in a CNN accelerator’s hidden layers is based on an optimization method utilizing a crossover function that changes weight values from primary to successor nodes constructed by zebra optimization. This optimization mimics the behaviour of zebra herd movement, where different candidate weights are selected based on their fitness. The fitness function applies this selection to guide the crossover process, ensuring that only the most effective weight combinations are carried forward. This selective updating reduces the number of unnecessary weight adjustments, leading to more efficient memory usage. This optimization improves the accelerator’s performance and memory usage. Moreover, the MOZC architecture is detailed in Fig 3.

MOZC neuron nodes vector calculation proceeds in the form of the neuron size, and for each *i*^th^ node, the vector calculus function parameter is determined as *y* , and *k* is the partial derivative variable. Hence, the vector model for the continuous chain process is described in Eq 3


$$\begin{array}{ccc} {\left[\frac{\partial y}{\partial k}\right]}_{i}=\frac{\partial y}{\partial z_{i}} & & \end{array}$$ (3)

The Loss function of the MOZC is executed by Eq 4 here, *p* is the specific target, *J*^*L*^ is the prediction outcome. Furthermore, the output phase of the CNN accelerator is built by Eq 5. Here, *G* denotes the output phase.


$$\begin{array}{ccc} {\text{loss}}_{i}=\frac{1}{2}{∥p-J^{L}∥}^{2} & & \end{array}$$ (4)

G= arg ⁡ i min ⁡ lossi (5)

The output in Eq 5 is obtained by reducing the loss. Here, *loss_i_* represents the loss calculated for each iteration. Minimizing this loss ensures that the predictions are continuously refined to converge toward the optimal target. Memory computing in the MOZC accelerator includes three prime parameters: control unit, cache, and arithmetic logic unit. Here, the optimization procedure is applied in the control unit to tune the processing elements.


$$\begin{array}{ccc} A_{c}(m)=X & & \end{array}$$ (6)

The accelerator is described as *A_c_*. The memory resource tuning is proceeded via Eq 6. If the memory utilization *m* does not meet the accelerator’s optimal utilization, then the accelerator parameters are further tuned to return the optimal status.


$$\begin{array}{ccc} N_{c}=h\{1,2,3,\ldots ,n\} & & \end{array}$$ (7)

The network communication constraints are determined as *N_c_* and *h* is the network hubs and *n* number of network nodes are determined as 1,2,3...*n*. The network node denotes a processing unit within the CNN architecture for data transportation, computation, and routing, particularly within the MOZC framework. Each network node plays a crucial role in processing information. It determines the shortest routing path by embedding a fitness function in the MOZC strategy and then supporting the data broadcasting procedure to validate network stability. The initialization of network communication constraints is defined in Eq 7. Once the Accelerator is designed with sufficient control features, the routing mechanism is incorporated for executing the data-sharing process. The routing parameters were designed in a single chip, based on the chip function, and the routing system was established to offer the routing mechanism.


$$\begin{array}{ccc} S_{p}=\{\begin{array}{cc} \text{nearest},\; & n_{p}<t\\ \text{distant},\; & n_{p}>t \end{array} & & \end{array}$$ (8)

In routing, the distance between each node from the source is measured after placing the nodes. Then, during the data-sharing process, the shortest route is determined to pass the message within a short duration, which can reduce communication delay. Hence, if the source node number is then the destination node number is 8, the path between the 2^nd^ and 8^th^ node was already determined during the shortest path selection; if the selected path distance is less than the predefined path, it is considered the nearest path. Otherwise, the shortest path selection process was continued. The function for determining the shortest route is exposed as *S_p_*. Here, t is the threshold of the destination, and the path length is determined as *n_p_*. If the path length is smaller than the decided threshold, it is considered the shortest distance. If the path distance is longer than the defined threshold, the path selection process is continued, and the shortest path is still found. Hence, the shortest route finding process is equated in Eq 8. The route determination is based on the zebra cross-section function equated in Eq 9.


$$\begin{array}{ccc} r_{h}=1-d^{*}\times \left(\frac{1}{D}\right) & & \end{array}$$ (9)

For finding the shortest path, the random neighbour node selection is valued by *r_h_* parameter. Then the current distance of the selected node from the source position is represented as d^*^ and the recorded distant route distance is denoted as *D*.

Two statistics parameters were obtained, *avg* and *std*, to quantify the executed algorithm, and they were evaluated based on Eq 10 and Eq 11.


$$\begin{array}{ccc} \text{avg}=\overset{S}{\underset{i=1}{\sum }}\frac{f}{s} & & \end{array}$$ (10)

$$\begin{array}{ccc} \text{std}=\sqrt{\frac{{\left(f-\text{avg}\right)}^{2}}{s}} & & \end{array}$$ (11)

Here, *f* determines the finest solution that is optimal for shortest path selection *s* is the iteration count to find the best path. Besides, the sensitivity range of the implemented accelerator is valued using Eq 12.


$$\begin{array}{ccc} g=\frac{{\text{avg}}_{1}-{\text{avg}}_{2}}{\sqrt{\frac{{\text{std}}_{1}^{2}+{\text{std}}_{2}^{2}}{s}}} & & \end{array}$$ (12)

Here, the *avg*_1_ and *avg*_2_ are attained from each iteration path finding to broadcast the data from one source end to another. Also, the *g* denotes the sensitivity range. All functioning models of MOZC are described in Algorithm 1. The Eqn notation with its description is tabulated in Table 1.

**Algorithm 1 MOZC algorithm.**



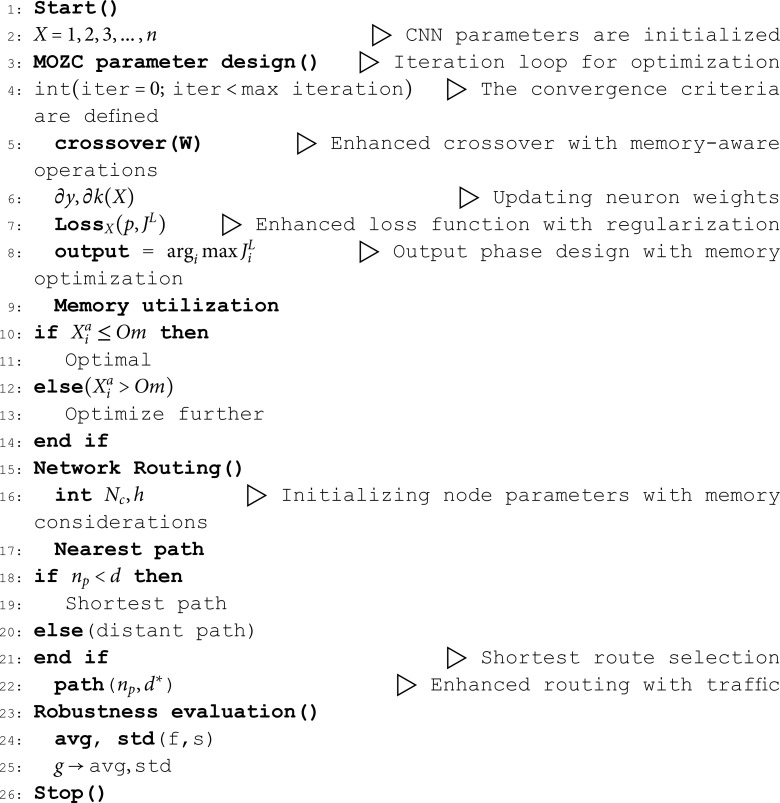



**Table 1:** Symbol description.

Symbol	Description
*X*	Input Data
*I*	Importing Data
*T*	Training Function
*W*	Weight Updation
*y*	Vector Calculus
*k*	Partial Derivative
*p*	Specific Target
*J* ^*L*^	Prediction Outcome
*G*	Output Phase
*A_c_*	Accelerator
*m*	Memory utilization
*N_c_*	Network Communication
*h*	Network Hub
*S_p_*	Shortest Path
*n_p_*	Path Length
*t*	Threshold
*r_h_*	Random neighbour Node
*d*	Source Position
*D*	Distant Route Distance
*f*	Fitness Function
*s*	Shortest Path Selection
*g*	Sensitivity Range

**Fig 4:**
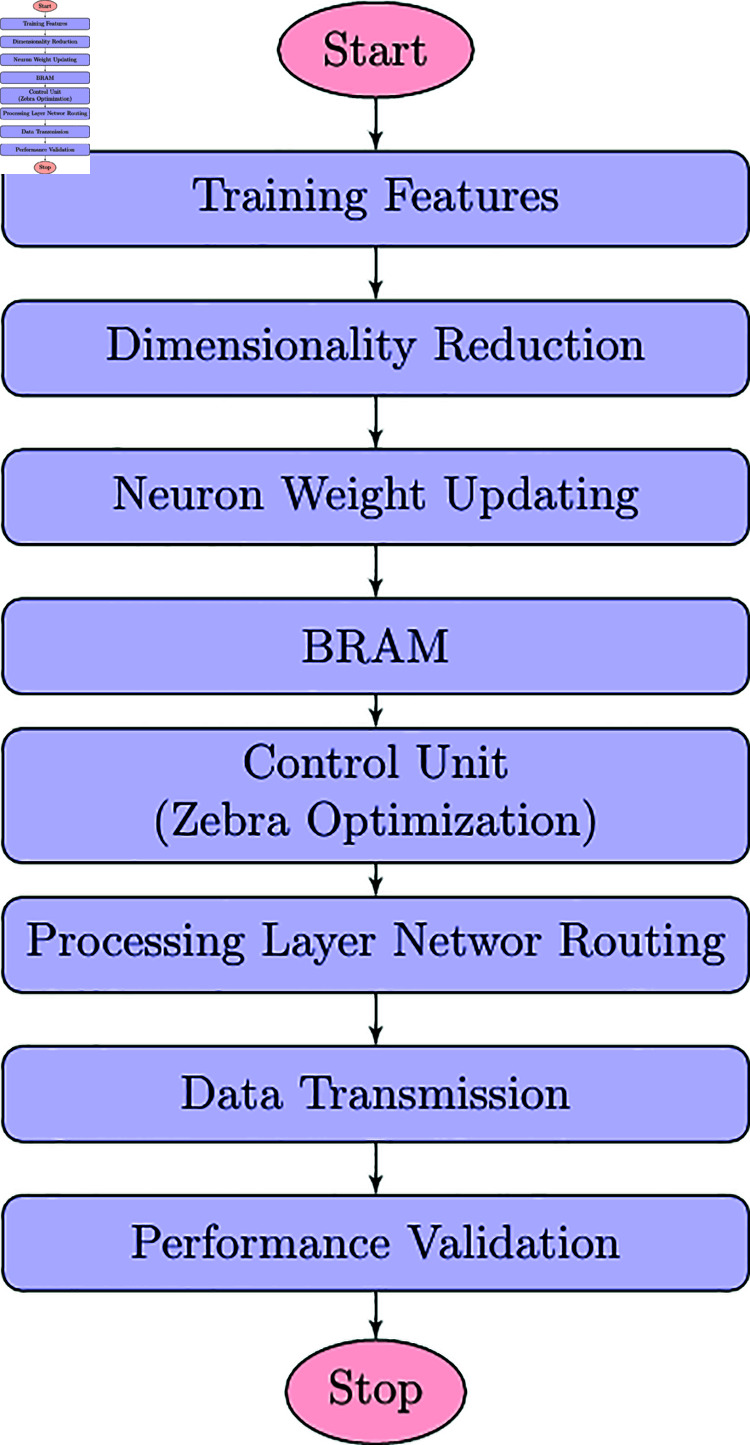
The workflow illustration of the proposed MOZC.

The complete flow of MOZC is established in Fig 4.

## 5 Results and discussion

The CNN accelerator is designed to improve network routing technologies and use CPU memory more effectively. The test parameters required that MOZC be run on this specialized Accelerator to measure its efficiency in routing networks and sharing data. Comparing the performance of an optimized CNN against traditional models displayed benefits in memory management as well as routing efficiency due to the careful tuning of functional accelerator parameters together with the DSR protocol. The results section was drawn for the robustness estimation of the built-optimized Accelerator. Here, the built Accelerator is tested in the Python environment, and the performance score is measured through the FPA module. Henceforth, the prime metrics were calculated by following specific mathematical steps, and the comparative validation was done with other traditional models. The execution elements of the built MOZC are tabulated in Table 2.

### 5.1 Case study

The National Football League impact detection dataset has been taken from the Kaggle site [37]. It has 9947 video frame images. The simulation parameters for the work are described in Table 3. In this simulation, MOZC was developed with key essential functional parameters to improve network routing efficiency; filter sizes used by convolutional layers are 3x3, Fully connected layers are 5x5, and pooling layers are 2x2. ReLU activation functions between the layers provide non-linearity and help learn complex patterns. Used 100 iterations of Zebra Optimization and also started a data broadcasting to confirm the stability of our framework.

**Fig 5:**
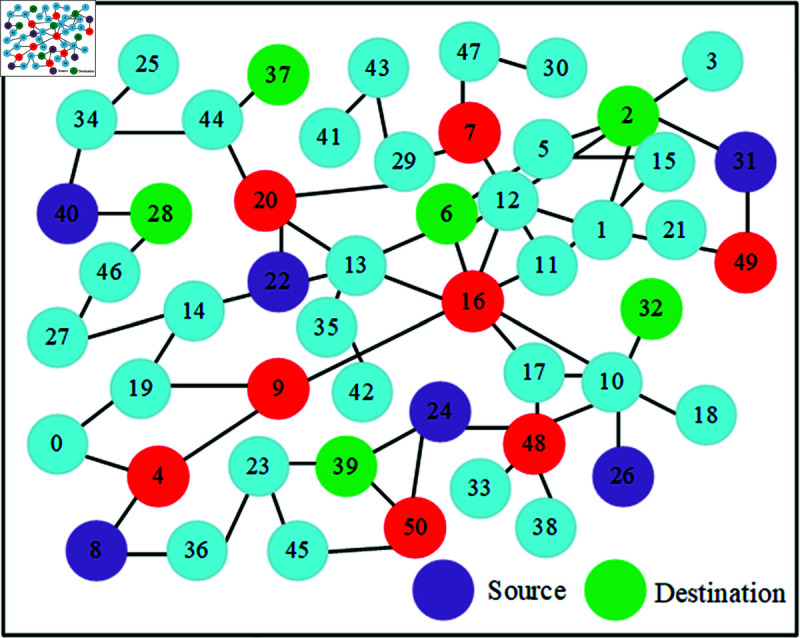
DSR routing.

To justify the designed accelerator performance, the DSR routing model was built, and the robustness was validated. Hence, the created routing network for forwarding the data between the users is displayed in Fig 5. It illustrates routing in the CNN accelerator optimization of a 20-node network. Nodes convey processing elements while the connections depict data flows in this network. The source and destination nodes, Red, Green and violet, are key elements for control or data routing on the Accelerator. This figure illustrates the neural network used internally by an accelerator, showing how data is moved around and optimized between different nodes to use memory efficiently for improved computation efficiency. It is a concept that visualizes the performance of zebra optimization by selecting optimal routes and connections while keeping memory overheads extremely low.

**Fig 6:**
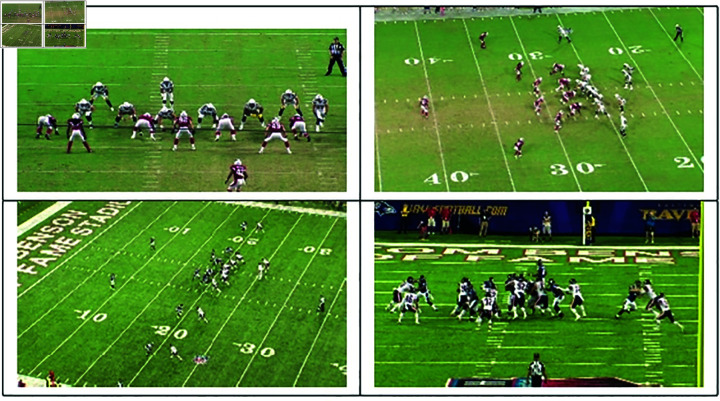
Samples of NFL dataset input video frames [37].

Simple DSR routing is enabled to validate the accelerator working scenario, and video data is considered for broadcasting purposes. Six nodes were created to generate the routing, the shortest path was found, and the data was broadcast. Also, as the input file, the video frame is taken and processed between the source and the destination. The sample input data is visualized in Fig 6

*Throughput and Packet Transfer Rate*: The parameter packet transfer and throughput score are considered when measuring the routing capacity. Hence, the recorded packet delivery range for the implemented replica is 95.5%, and the conventional CNN has reported a 70.1 packet transfer rate. In addition, the traditional CNN has scored 71.2 Mbps throughput, and the presented MOZC routing accelerator has gained 96.4 Mbps. Hence, the packet broadcasting is analyzed by Eq 13.


$$\begin{array}{ccc} \text{packet transfer}=\frac{\text{packet received}}{\text{total packet sent}} & & \end{array}$$ (13)

**Table 2:** Execution constraints.

Device Constraints	
Language	Python
RAM	16GB
Package	Tensorflow and Pytorch
CPU	3.2 GHz
Processor	Intel Core i5
Operating System	Win 10
Application	Network Routing
Training Accelerator	CNN
Optimization	Zebra
FPGA device	Xilinx Virtex 7

The FPGA performance recorded to make the routing process is visualized in Table 4. The following Table summarizes the improvements made using the MOZC optimization technique. In particular, the optimization resulted in a significant reduction in flip-flops (FF) and look-up tables (LUT), with an increased utilization of digital signal processing (DSP). More DSP usage describes MOZC using more DSP blocks for computations, which shows better performance and efficiency by reducing the computational load. The results show that the optimization method can optimize resource utilization and improve CNN accelerator performance. Despite having dissimilar characteristics, the different resources available before and after optimisation are executed in FPGA boards because of optimization methods and resource allocation strategies. Besides, some prime parameters were valued for the conventional CNN and the Proposed MOZC to measure CNN’s rapidity in training and testing. Those metrics descriptions and the performance range are tabulated in Table 5. These metrics are derived from extensive testing. The performance of the accelerator was characterized by measuring both memory usage and how effectively it managed memory resources during CNN processing. To facilitate the measurement of execution time, inference tasks on CNNs are being accelerated by FPGA devices. The amount of power taken while running the Accelerator is observed to check power utilisation. Latency was determined by the time delay between input-output response time, and GOPS/w is the number of operations performed for each watt of power consumed. These metrics have been compared for both a conventional CNN and the introduced MOZC accelerator, which shows the gain in efficiency due to these optimization techniques.

**Table 3:** Simulation parameters.

Parameters	Values
Convolution Layer	5
Fully Connected Layer	2
Max Pooling Layer	2
Filter Size	3x3,5x5,2x2
Activation Function	ReLu
Iteration	100

**Table 4:** FPGA device performance.

Device Performance						
FPGA Device Parameters	Before Optimization CNN			After Optimization MOZC		
	Available	Utilization	Utilization (%)	Available	Utilization	Utilization (%)
**FF**	2485582	89641	3.6	2804582	51452	1.83
**LUT**	1685582	85478	5.07	1504582	43485	2.89
**DSP**	8122	1212	14.92	8045	2585	32.13
**BRAM**	3592	2980	82.96	2992	2324	77.67

**Table 5:** MOZC performance.

Accelerator Performance Constraints	CNN	Proposed (MOZC)
Memory Usage (MB)	3201.05	2984.55
Execution Time (ms)	28.58	19.75
Power Utilization (W)	110.42	46.47
Latency (micro sec)	105.71	12.15
GOPS	9.34	30.43

Throughput is measured in Megabits per second (Mbps). It quantifies the rate at which data is transmitted or processed within a system. In contrast, efficiency is expressed as GOPS per Watt (GOPS/W). It measures the system’s computational operations, representing the number of operations carried out for each watt. Table 5 shows MOZC performance. The increase in efficiency to Latency demonstrates that MOZC achieves an excellent execution time reduction without losing performance concerning operations per second. This describes that the MOZC can accomplish more quickly by being faster and reacting quicker, potentially with optimized power usage.

***Power***: The energy resources needed to execute the accelerator function are evaluated as energy usage. The energy utilization should be optimal to earn the finest FPGA device functioning rate. Besides, the energy utilization parameter is the chief metric for evaluating the accelerator performance.

### 5.2 Performance analysis

Some prime metrics were employed to quantify the presented novel MOZC accelerator, and a comparison was made with the traditional approaches. Hence, the performance constraints are memory usage, FF, LUT, and DSP. Also, the previously implemented methods that are utilized for this performance validation are the Mimosa pudica Convolution Neural (MPCN) model, Open-source Optimized Accelerator (OOA) [28], Compressive-Depth Accelerator (CA) [29], and Linear Erosion Framework (LEF) [30]. The MPCN uses the Mimosa pudica plant’s uniqueness in sensitivity properties to improve the feature extraction processes by hybridizing with the CNN. As a result, the proposed approach has comprehensive efficiency metrics for the usage of resources. The OOA model achieves flexible and improved performance on FPGA design parameters. It achieves efficient speed up and efficiency. The CA model uses an ADMM solver to optimize the resources, demonstrating that fewer resources are utilized. LEF offers feasible and scalable applications. It minimizes the speed and resource usage.

**Fig 7:**
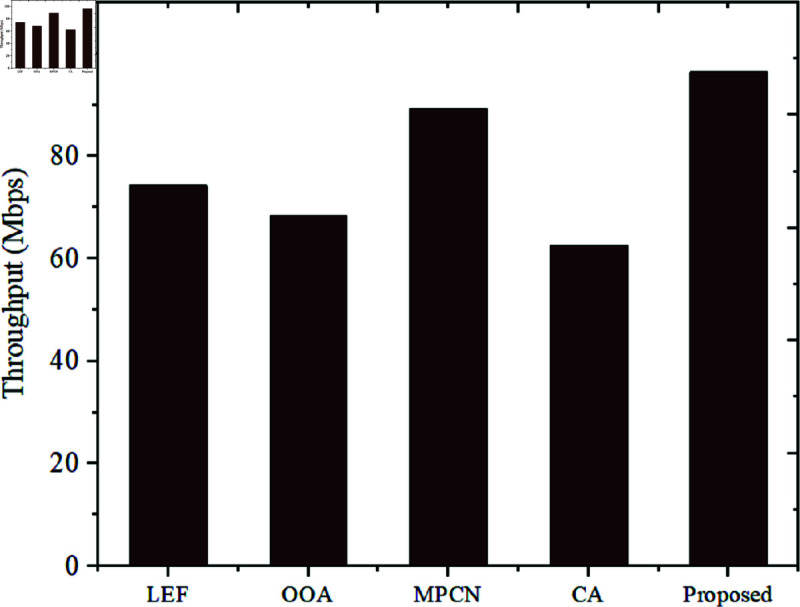
Throughput comparison.

**Fig 8:**
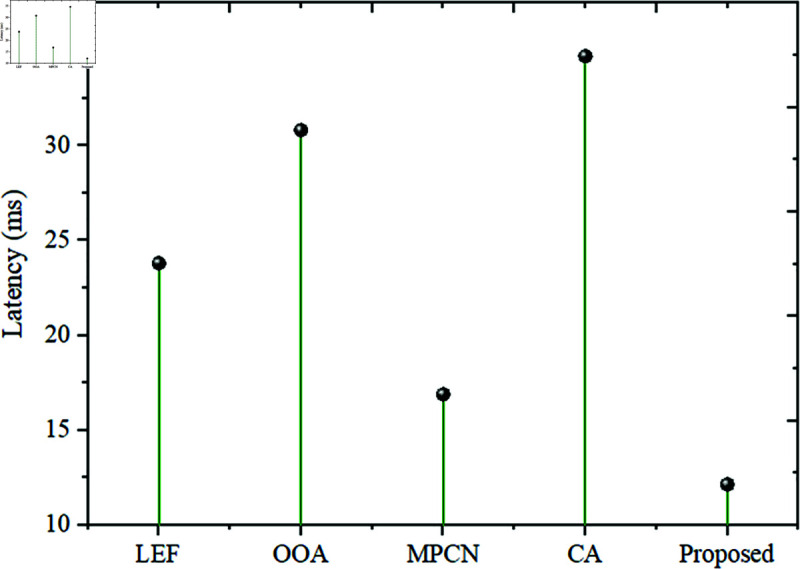
Latency comparison.

To compare the Throughput and Latency of the proposed model, Throughput and Latency metrics are assessed for the existing techniques such as LEF, OOA, MPCN, and CA and the comparison is shown in Fig 7 and Fig 8.

The Throughput rate attained by the existing LEF is 74.3 Mbps, OOA is 68.4 Mbps, MPCN is 89.2 Mbps, and CA is 62.6 Mbps. The developed model performs better than the existing techniques by attaining a high throughput of 96.4 Mbps. This shows that the data is more efficiently transmitted due to better use of resources.

***Latency***: Here, the Latency is measured in the routing strategy case; the duration needed to broadcast the data between sender and receiver is valued as Latency. The latency parameter helps to analyze the Accelerator’s rapidity performance.

The Latency gained by the existing LEF is 23.8 ms, OOA is 30.8 ms, MPCN is 16.9 ms, and CA is 34.7 ms. The proposed model gained a low latency of 12.15 ms. It shows that the proposed is the fastest compared to all other methods. Therefore, it results in a more rapid delivery of data packets transmitted using the proposed method than other approaches.

***Memory usage***: The required space for the built-optimized CNN accelerator is measured as memory usage. If the Accelerator has consumed more space, then high energy intake is reported. Besides, the increased memory utilization might degrade the accelerator performance. Based on the memory usage scenario, BRAM is measured.

**Fig 9:**
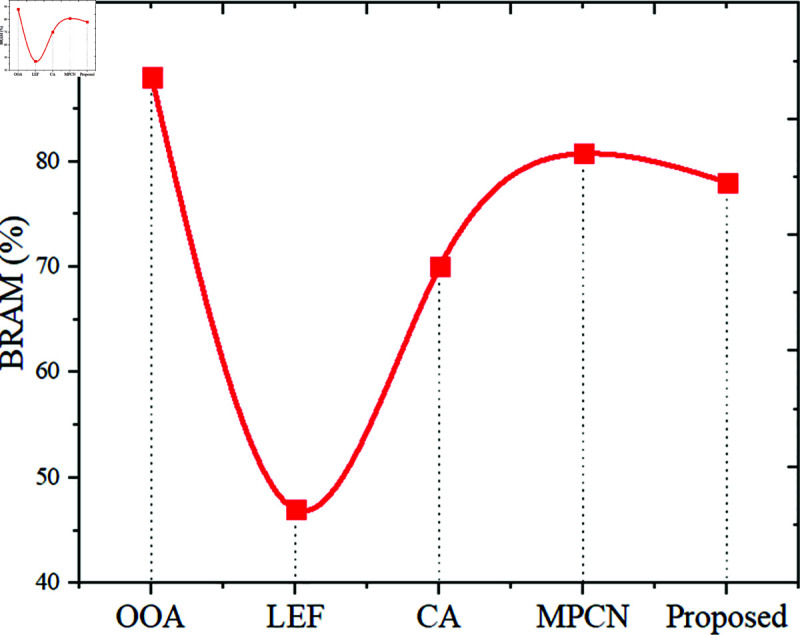
BRAM performance.

The LEF model reported BRAM usage as 47%, OOA utilized 88% BRAM, MPCN utilized 80.75% BRAM, and the CA model used 70% BRAM. Considering all these strategies, the implemented Accelerator MOZC scored 78 in BRAM utilization, displayed in Fig 9. The proposed MOZC model has a better trade-off between resource efficiency and performance, which means that the embedded CAN modules consume less BRAMs compared to OOA and MPCN. It offers improved performance due to optimized architecture and algorithmic design but does not beat LEF because it uses specific memory optimization methods.

***Flip-Flop and LUT***: The prime factors, Flip-flop or LUT, are measured to store the data and process it to the following blocks. Hence, these two prime components are the FPGA module’s data storage element. The high storing capacity and the stableness might tend to earn the vast LUT and FF scores.

**Fig 10:**
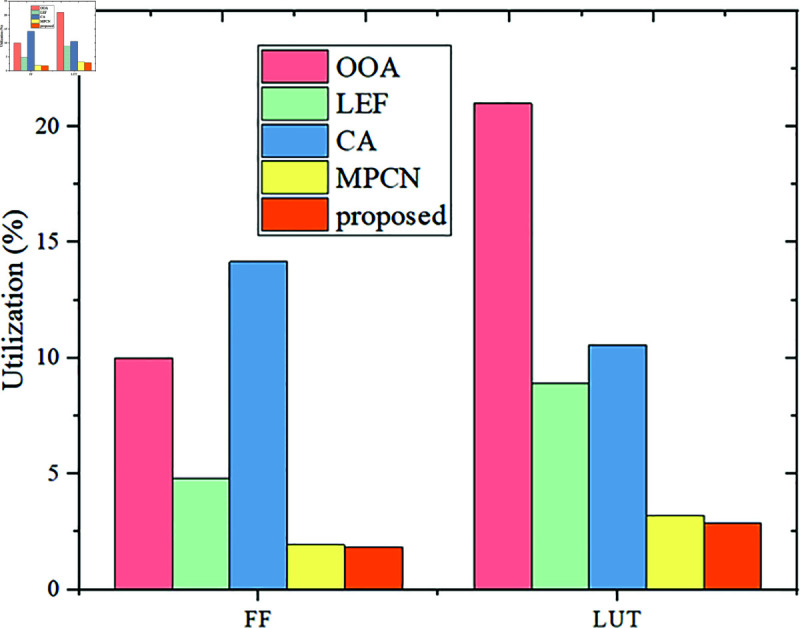
FF and LUT performance.

Here, the LEF strategy scored 4.8% FF utilization, OOA reported 10% FF usage, MPCN earned 1.94% FF, and the model CA earned 14.2% FF usage. Compared to these traditional subjects, the novel MOZC has utilized 1.83% FF usage. Besides, the utilized LUT by the model LEF is 8.9%, OOA utilized 21% LUT, MPCN utilized 3.26% LUT, CA utilized 10.6%, and the novel MOZC scored 2.89% LUT, it is because of the optimized Accelerator, explored in Fig 10. The model proposed finds a trade-off between resource and implementation efficiency. It achieves lower FF and LUT utilization than OOA and MPCN, proving its denser logic resource efficiency for both methods.

**Fig 11:**
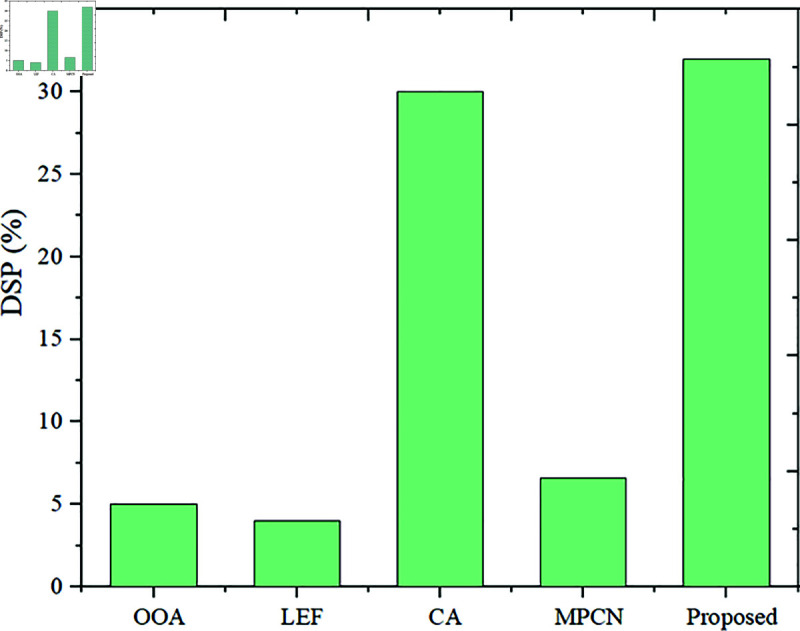
DSP utilization.

***DSP***: The DSP utilization of the LEF approach is 4%, and the percentage of DSP utilization by the OOA model is 5%. Moreover, the CA approach reported the DSP utilization as 30%, as explained in Fig 11. Considering this, the novel accelerator has scored the DSP utilization score as 32%, which is better than other traditional approaches. It is more hardware-resource efficient than OOA, LEF, and MPCN in DSP utilization. MOZC performs better than OOA, LEF, and MPCN due to its efficient architecture and algorithm design, which assumes weight sharing. The memory usage and efficiency attained by the existing LEF is 2993.43 and 21.8, OOA is 3054.23 and 14.7, MPCN is 3246.7 and 29.3, CA is 3498.23 and 24.9 and the proposed model attained 2984.55 and 30.43 respectively. It proves the proposed model performs better by obtaining low memory usage and high efficiency.

**Table 6:** Comparison statistics.

Comparison					
Device Parameters	LEF	OOA	MPCN	CA	Proposed
**FF(%)**	4.8	10	1.94	14.2	1.83
**DSP(%)**	4	5	6.78	30	32
**LUT(%)**	8.9	21	3.26	10.6	2.89
**BRAM(%)**	47	88	80.75	70	78
**Throughput(Mbps)**	74.3	68.4	89.2	62.6	96.4
**Latency(ms)**	23.8	30.8	16.9	34.7	12.15
**Memory Usage(MB)**	2993.43	3054.23	3246.7	3498.23	2984.55
**Efficiency (GOPS/W)**	21.8	14.7	29.3	24.9	30.43

The proposed method enhances the performances by reducing 0.2% flip-flop and 2% in LUT usage, 2% improvement in the DSP, 8% improvement in Throughput, and 7% and 2% reduction in latency and memory usage, respectively, and efficiency is increased to 3% than the existing approaches. Considering all FPGA and routing performance, the implemented Accelerator has earned an outstanding performance measure, which indicates the robustness of the executed MOZC accelerator for the routing application Table 6. The MOZC improves the efficiency of the CNN accelerator while minimizing resource utilization, particularly CPU memory usage. The design focuses on reducing the computational overload by optimizing the CNN architecture and selecting efficient filter sizes. The zebra optimization algorithm is employed to fine-tune the Accelerator’s performance by adjusting key hyperparameters such as crossover rate and iterations, leading to faster convergence with minimal hardware demand. A robust network routing mechanism was integrated into the design to assess the effectiveness of the Accelerator, ensuring that the system can handle routing tasks efficiently without overloading memory resources. This process results in both performance efficiency and resource usage.

Additionally, to analyse the hybrid of Zebra optimization (ZO) in CNN, the proposed model’s performance is compared with hybridizing CNN with other optimizations such as Particle swarm (PS), Grey Wolf (GW), Ant Colony (AC) and spider wasp (SW) optimization. The metrics such as LUT, FF, DSP, BRAM, Throughput, Latency, memory usage and Efficiency are evaluated, and the results are displayed in Table 7.

**Table 7:** Neural network with optimization comparison.

Comparison					
Device Parameters	CNN+PS	CNN+GW	CNN+AC	CNN+SW	CNN+ZO Proposed
LUT (%)	4.8	3.49	3.69	5.8	2.89
FF (%)	6.71	9.56	3.89	2.98	1.83
DSP (%)	42	37	40	39	32
BRAM (%)	69	71	63	54	78
Throughput (Mbps)	87.2	84.3	70.9	81.4	96.4
Latency (ms)	19.7	21.5	27.6	16.6	12.15
Memory usage (MB)	3200.71	4192.82	4582.12	3892.61	2984.55
Efficiency (GOPS/w)	29.8	16.8	21.5	19.7	30.43

Table 7 presents the benefits of the developed model that uses ZO and CNN compared to other optimization algorithms like PS, GW, AC, and SW. The ZO-based model results in optimal performance across the considered metrics. It presents the lowest resource utilisation, indicating efficient hardware resource usage. This efficiency is coupled with notable performance gains, including the highest Throughput and lowest Latency, critical metrics in high-performance computing applications. Additionally, memory usage is the lowest among the methods compared, which is crucial for scalability and power efficiency. Moreover, ZO demonstrates the highest efficiency in terms of computational performance, significantly outperforming the other methods. These results highlight the novel and efficient ZO in the optimization tasks of CNNs.

Moreover, to analyze the MOZC performance against other neural networks, the ZO is combined with recurrent neural network (RNN), Fully convolutional network (FCN), Residual Network (RSN) and Capsule Network (CSN), and the results are displayed in Table 8

**Table 8:** Optimization with neural network comparison.

Comparison					
Device parameters	ZO+RNN	ZO+FCN	ZO+RSN	ZO+CSN	ZO+CNN Proposed
LUT (%)	5.9	4.56	4.78	3.56	2.89
FF (%)	7.81	8.75	3.26	3.09	1.83
DSP (%)	53	44	39	41	32
BRAM (%)	66	70	65	72	78
Throughput (Mbps)	77.1	89.4	71.6	80.7	96.4
Latency (ms)	26.7	21.7	28.9	20.8	12.15
Memory Usage (MB)	4301.82	3917.62	4768.91	3768.91	2984.55
Efficiency (GOPS/w)	25.8	21.7	15.8	29.8	30.43

The results in Table 8 describe the performance of the proposed model by hybridizing CNN with optimization performance better than hybridising with other neural networks. It provides the most efficient performance in all key metrics.

***Applicability***: The developed MOZC model has a specific filter size parameter that was made to maximize the performance of the MOZC framework for the particular application. However, the proposed methodology, including ReLU activation functions, Zebra Optimization, and data broadcasting, is not specified in this architecture and may be adapted to other deep neural network (DNN) architectures. The essential functional parameters, such as filter sizes or layer types, may be changed to accommodate alternative architectures without affecting the fundamental optimisation process or stability confirmation. The Zebra Optimisation approach, which is flexible and adaptable, can improve efficiency in any DNN framework. These components help enhance routing efficiency and stability, irrespective of the specific layer sizes or configurations.

## 6 Conclusion

Digitalized communication is broadly developed in many innovative scenarios, and communication strategy functioning performance was based on the routed chip module of the network accelerator. Considering this advancement, a novel MOZC accelerator is implemented in Python using NFL dataset 9947 video frame images. Hence, the routing application is executed with the DSR protocol to value the accelerator efficiency. By performing the routing standard, the implemented MOZC optimized memory usage and obtained 2984.55 MB by reducing 2% more than the previous studies. Hence, the reported BRAM utilization rate is 78%, which is a higher utilization rate than traditional approaches. The reported FF utilization is 1.83%. Compared to another conventional model, 0.2% of FF usage was minimized. The Throughput is 96.4%, which shows an 8% improvement; the Latency is 12.5 ms, showing a 7% reduction in Latency, and the efficiency is 30.43 GOPS/w increased to 3% than the existing approaches. Moreover, the utilization percentage of LUT is 2.89%; compared to the past models, 1% of LUT utilization was minimized. This optimal status has verified that the built Accelerator is highly adaptable for the routing application. However, this model lacks security; the routing model is always vulnerable to attack. Despite the lack of implementation of the security paradigm, the authenticity remains uncertain. Hence, future work is needed to implement the security accelerator for the routing application, which could provide a better outcome.
